# Supplementary material for: Clinical Outcomes of Online Adaptive Magnetic Resonance-Guided Stereotactic Body Radiotherapy of Adrenal Metastases from a Single Institution
Source: Cancers (Basel). 2024 Jun 19;16(12):2273. doi: 10.3390/cancers16122273 (PMC11201609; doi:10.3390/cancers16122273)
Supplement: Supplementary file 1 [file cancers-16-02273-s001.zip › cancers-3000698-supplementary.pdf]

cancers

Table S1. Systemic therapy three months prior to and six months after adrenal SBRT.

	n
Systemic therapy 3 months prior to SBRT	
Yes	25
No	12
Agents	
Pemetrexed+Pembrolizumab	5
Pemetrexed	1
Cisplatin+Vinorelbine	1
Cisplatin+5-fluouruacil	1
Cisplatin+5-fluouruacil+Pembrolizumab	1
Carboplatin+Vinorelbine	1
Carboplatin+Etoposide+Atezolizumab	1
Carboplatin+nab-Paclitaxel	1
Carboplatin+nab-Paclitaxel+Pembrolizumab	2
Docetaxel	1
Azacitidine+Venetoclax	1
Nivolumab	2
Pembrolizumab	2
Atezolizumab	2
Sunitinib	1
Trametinib	1
Tumor vaccination	1
Systemic therapy 6 months after SBRT	
Yes	24
No	13
Agents	
Cisplatin+Vinorelbine	1
Cisplatin+5-fluouruacil+Pembrolizumab	1
Carboplatin+Vinorelbine	1
Carboplatin+nab-Paclitaxel	1
Gemcitabine+nab-Paclitaxel	1
Pemetrexed+Pembrolizumab	5
Paclitaxel+Ramucirumab	1
Atezolizumab	3
Nivolumab	2
Pembrolizumab	3
Ipilimumab	1
Lenvatinib+Everolimus	1
Mekinist	1
Sunitinib	1
Bevacizumab	1
